# IL-6 regulation on skeletal muscle mitochondrial remodeling during cancer cachexia in the *Apc*^*Min/+*^ mouse

**DOI:** 10.1186/2044-5040-2-14

**Published:** 2012-07-06

**Authors:** James P White, Melissa J Puppa, Shuichi Sato, Song Gao, Robert L Price, John W Baynes, Matthew C Kostek, Lydia E Matesic, James A Carson

**Affiliations:** 1 Integrative Muscle Biology Laboratory, Exercise Science Department, Columbia, SC, USA; 2 Department of Biological Sciences, Columbia, SC, USA; 3 Cell and Developmental Biology and Anatomy, USC School of Medicine, University of South Carolina, Columbia, SC, USA; 4 University of South Carolina, Department of Exercise Science Public Health Research Center, 921 Assembly St., Room 405, Columbia, SC, 29208, USA

**Keywords:** FIS1, PGC-1α, Exercise, IL-6r, MFN1, Cachexia, Mitochondria, Muscle, Autophagy

## Abstract

**Background:**

Muscle protein turnover regulation during cancer cachexia is being rapidly defined, and skeletal muscle mitochondria function appears coupled to processes regulating muscle wasting. Skeletal muscle oxidative capacity and the expression of proteins regulating mitochondrial biogenesis and dynamics are disrupted in severely cachectic *Apc*^*Min/+*^ mice. It has not been determined if these changes occur at the onset of cachexia and are necessary for the progression of muscle wasting. Exercise and anti-cytokine therapies have proven effective in preventing cachexia development in tumor bearing mice, while their effect on mitochondrial content, biogenesis and dynamics is not well understood. The purposes of this study were to 1) determine IL-6 regulation on mitochondrial remodeling/dysfunction during the progression of cancer cachexia and 2) to determine if exercise training can attenuate mitochondrial dysfunction and the induction of proteolytic pathways during IL-6 induced cancer cachexia.

**Methods:**

*Apc*^*Min/+*^ mice were examined during the progression of cachexia, after systemic interleukin (IL)-6r antibody treatment, or after IL-6 over-expression with or without exercise. Direct effects of IL-6 on mitochondrial remodeling were examined in cultured C2C12 myoblasts.

**Results:**

Mitochondrial content was not reduced during the initial development of cachexia, while muscle PGC-1α and fusion (Mfn1, Mfn2) protein expression was repressed. With progressive weight loss mitochondrial content decreased, PGC-1α and fusion proteins were further suppressed, and fission protein (FIS1) was induced. IL-6 receptor antibody administration after the onset of cachexia improved mitochondrial content, PGC-1α, Mfn1/Mfn2 and FIS1 protein expression. IL-6 over-expression in pre-cachectic mice accelerated body weight loss and muscle wasting, without reducing mitochondrial content, while PGC-1α and Mfn1/Mfn2 protein expression was suppressed and FIS1 protein expression induced. Exercise normalized these IL-6 induced effects. C2C12 myotubes administered IL-6 had increased FIS1 protein expression, increased oxidative stress, and reduced PGC-1α gene expression without altered mitochondrial protein expression.

**Conclusions:**

Altered expression of proteins regulating mitochondrial biogenesis and fusion are early events in the initiation of cachexia regulated by IL-6, which precede the loss of muscle mitochondrial content. Furthermore, IL-6 induced mitochondrial remodeling and proteolysis can be rescued with moderate exercise training even in the presence of high circulating IL-6 levels.

## Background

Although the regulation of muscle protein turnover during cachexia is rapidly being defined and the importance of protein degradation processes is clearly demonstrated, questions remain related to the underlying physiological drivers that initiate alterations in these processes throughout the progression of cachexia. There is accumulating scientific support for differential mechanisms contributing to muscle loss during transition from the initiation of cachexia toward severe cachexia [1,2]. We have recently described differential regulation of muscle protein turnover between the initial stages of cachexia and severe body weight loss in the *Apc*^*Min/+*^ mouse [1]. With wasting conditions, a reduction in aerobic capacity is clearly associated with the degree of body weight and muscle mass loss [3,4]. Emerging evidence also provides for a role of muscle mitochondria in the regulation of muscle protein turnover [5]. Mitochondrial dynamics and biogenesis are sensitive to contractile activity, in particular endurance based exercise [6,7]; however, the underlying mechanisms governing these processes during conditions of skeletal muscle wasting remain poorly defined.

The coordinated balance between mitochondrial fission and fusion, referred to as mitochondria dynamics [8], and muscle protein degradation have been described by Romanello and Sandri [5]. The proposed model suggests mitochondrial dysfunction results in reactive oxidative species, susceptibility to apoptosis and energy stress. These processes can lead to downstream activation of muscle proteolytic activation through AMPK and FoxO activation [5]. Previous reports from our laboratory have shown cachectic *Apc*^*Min/+*^ mice to have reduced muscle mitochondrial content associated with increased apoptosis, suppression of the peroxisome proliferator-activated receptor-gamma co-activator 1 alpha (PGC-1α) and altered regulation of mitochondrial fission and fusion independent of oxidative stress [9,10]. In addition, we have recently shown increased activation of AMPK and FoxO in muscle from severely cachectic *Apc*^*Min/+*^ mice [1]. The increase in fission and decrease in PGC-1α and mitochondrial fusion during cachexia has been previously reported [9]; however, it is not known if these alterations are early events in the onset of muscle wasting, and have a regulatory role in the progression of cachexia.

Inflammatory cytokines are well-established mediators of muscle wasting during cancer cachexia [11,12], and anti-cytokine treatments can attenuate cachexia progression [1,13,14]. In the *Apc*^*Min/+*^ mouse model of cachexia, the cytokine IL-6 is necessary for muscle wasting [15], and over-expression of circulating IL-6 in precachectic *Apc*^*Min/+*^ mice accelerates the development of cachexia [16]. We have recently reported that IL-6 receptor (IL-6r) antibody administration to cachectic *Apc*^*Min/+*^ mice attenuates further progression of cachexia [1], and was associated with suppressed muscle protein degradation. IL-6r antibody administration also represses lysosomal and autophagy-related protein expression in cachectic muscle [1,14]. There is supporting evidence for direct effects of IL-6 on muscle mitochondrial dynamics as treating cultured human myoblasts with IL-6 results in a reduction in the mitochondrial fusion protein Mfn2 [17]. However, IL-6 regulation of muscle mitochondrial remodeling during the progression of cachexia is not clear and warrants further attention.

Endurance exercise training increases skeletal muscle oxidative capacity [6,7] and has been effectively used as a counter measure for numerous muscle wasting conditions, including diabetes [18], chronic obstructive pulmonary disease (COPD) [19], renal disease [20] and cardiac cachexia [21]. We have recently found that IL-6 over-expression-induced bodyweight and muscle mass loss in *Apc*^*Min/+*^ mice is prevented by moderate treadmill exercise, and is associated with an induction of muscle oxidative protein expression [22]. Due to these findings, the current study has pursued an enhanced understanding of how mitochondrial content, biogenesis and dynamics are regulated during the progression of cachexia by IL-6. The purposes of this study were to 1) determine IL-6 regulation on mitochondrial remodeling/dysfunction and the subsequent induction of muscle proteolysis observed during the progression of cancer cachexia and 2) to determine if exercise training can attenuate mitochondrial dysfunction and the induction of proteolytic pathways during IL-6 induced cancer cachexia. We hypothesized the altered expression of muscle proteins regulating mitochondria biogenesis, fission and fusion would be regulated by IL-6 in muscle at the onset of cachexia and precede mitochondrial content loss, which is most prominent during late stage cachexia. We also hypothesized that exercise training would suppress IL-6-induced changes in mitochondria biogenesis, fission and fusion and, in turn, inhibit the induction of muscle proteolytic activation and muscle wasting.

## Methods

### Animals

*Apc*^*Min/+*^ mice on a C57Bl/6 background were originally purchased from Jackson Laboratories (Bar Harbor, ME, USA) and bred at the University of South Carolina’s animal resource facility as previously described [23]. *Apc*^*Min/+*^ (n = 21) mice were group housed and were sacrificed at various time points to provide stratification of body weight loss to study regulation of muscle mitochondrial remodeling during the progression of cachexia. The groups were as follows; weight stable (WS), <5% (initial), 9 to 16% (intermediate) and >20% (severe) cachexia. To block the progression of cachexia, a subset of *Apc*^*Min/+*^ mice were treated with an IL-6 receptor antibody (n = 5) or phosphate-buffered saline (PBS) control (n = 7) at 16 weeks after the onset of cachexia (See procedure below). Wild-type controls were also treated with the IL-6 receptor antibody (n = 6) or PBS control (n = 6) at 16 weeks. To increase circulating IL-6 levels, wild-type and *Apc*^*Min/+*^ mice (Control; n = 5 and + IL-6; n = 6) were used for IL-6 over-expression experiments (See procedure below). A subset of wild-type and *Apc*^*Min/+*^ mice were exercised (See exercise methods) or served as cage controls. The room was maintained on a 12:12 light:dark cycle with the light period starting at 0700. Mice were provided standard rodent chow (Harlan Teklad Rodent Diet, #8604, Madison, WI, USA) and water ad libitum. Body weight and food intake were measured weekly. All animal experimentation was approved by the University of South Carolina’s Institutional Animal Care and Use Committee.

### IL-6 receptor antibody administration

The MR16-1 IL-6 receptor antibody was a generous gift from Chugai Pharmaceutical Co., LTD, Tokyo, Japan. A total of 300 μg/mouse of IL-6 receptor antibody was administered by an intraperitoneal injection every 3 days for 2 weeks starting at 16 weeks of age. PBS was injected as a control vehicle.

### IL-6 over-expression

In vivo intramuscular electroporation of an IL-6 plasmid was used to increase circulating IL-6 levels in mice as previously described [17,24]. The quadricep muscle was used as a vessel to produce IL-6 and secrete it into circulation, and was not used for any analyses in this study. The gastrocnemius muscle used in the study was not subjected to electroporation. Briefly, mice were injected with 50 μg of the IL-6 plasmid driven by the CMV promoter, or empty control vector, into the quadriceps muscle. Mice were anesthetized with a 2% mixture of isoflurane and oxygen (1 L/minute). The leg was shaved, and a small incision was made over the quadricep muscle. Fat was dissected away from the muscle, and the plasmids were injected in a 50-μl volume of PBS. A series of eight 50 ms, 100 V pulses was used to promote uptake of the plasmid into myofibers, and then the incision was closed with a wound clip. Both vector control and + IL-6 groups received the appropriate plasmid starting at 12 weeks of age. Mice were killed after two weeks of IL-6 over expression.

### Plasma IL-6

Plasma IL-6 levels were measured with a mouse-specific ELISA (Biosource, Carlsbad, CA, USA) as previously described [17]. Blood samples were taken under brief isoflurane anesthesia from the retro-orbital eye sinus two weeks after electroporation to determine plasma IL-6 concentrations.

### Treadmill protocol

At five weeks of age, mice were grouped into either exercise (n = 16) or cage control (n = 20) at which time they started their training as previously described [22]. Briefly, acclimation consisted of running at a 5% grade for a total of 20 minutes with gradual increase in speed starting at 10 m/minute and increasing to 18 m/minute. After the three days of acclimation mice, started on a training regimen that consisted of a 5-minute warm up at 10 m/minute at 5% grade followed by 55 minutes of running at 18 m/minute at 5% grade. Mice were encouraged to run by gentle taps. Mice ran six days a week and were given one day of recovery. After electroporation at 12 weeks, the mice received a two-day break from exercise before starting again. Mice ran until 14 weeks of age when they were sacrificed.

### Tissue collection

Mice were given a subcutaneous injection of ketamine/xylazine/acepromazine cocktail (1.4 ml/kg BW) before the gastrocnemius was dissected. The gastrocnemius muscles were rinsed in PBS, weighed, snap frozen in liquid nitrogen, and stored at −80°C until further analysis.

### mtDNA PCR

Mitochondrial capacity was performed as previously described [9]. DNA was isolated using DNAzol® Reagent (Invitrogen, Carlsbad, CA, USA). Briefly, muscle (20 to 30 mg) was homogenized in 1 ml DNAzol, pelleted with 100% ethanol, and re-suspended in 8 mM NaOH. Quantitative real-time PCR analysis was carried out in 25 μl reactions consisting of 2x SYBR green PCR buffer (AmpliTaq Gold DNA Polymerase, Buffer, dNTP mix, AmpErase UNG, MgCl_2_) (Applied Biosystems, Foster City, CA, USA), 0.150 μg DNA, DI water, and 60 nM of each primer. PCR was run with the DNA sample with Cytochrome B Forward, 5′ - ATT CCT TCA TGT CGG ACG AG −3′; Cytochrome B Reverse, 5′ - ACT GAG AAG CCC CCT CAA AT - 3′, Gapdh Forward, 5′ - TTG GGT TGT ACA TCC AAG CA - 3′; Gapdh Reverse, 5′ - CAA GAA ACA GGG GAG CTG AG - 3′. Samples were analyzed on an ABI 7300 Sequence Detection System. Reactions were incubated for 2 minutes at 50°C and 10 minutes at 95°C, followed by 40 cycles consisting of a 15-s denaturing step at 95°C and 1-minute annealing/extending step at 60°C. Data were analyzed by ABI software (Applied Biosystems, Foster City, CA, USA). using the cycle threshold (C_T_), which is the cycle number at which the fluorescence emission is midway between detection and saturation of the reaction. The 2^-ΔΔ CT^ method [25] was used to determine changes in gene expression between Cytochrome B with Gapdh C_T_ as the correction factor. The ratio between mtDNA and nuclear DNA genes was normalized to weight stable *Apc*^*Min/+*^ mice and wild-type PBS treated mice and used as an index of mitochondrial content. This method has been modified from a previously used technique to determine mitochondrial content in muscle [26].

### C2C12 cell culture

C2C12 myoblasts purchased from American Type Culture Collection (Manassas, VA, USA) were cultured in Dulbecco’s modified Eagle’s medium (DMEM), supplemented with 10% FBS, 50 U/ml penicillin and 50 μg/ml streptomycin (Fisher Scientific, Pittsburg, PA, USA). Upon reaching confluence, myoblast differentiation was induced for 72 h in DMEM supplemented with 2% heat-inactivated horse serum (HIHS), 50U/ml penicillin and 50 μg/ml streptomycin. After 72 h differentiation, IL-6 (Sigma, St. Louis, MO, USA) was added to serum-free DMEM and incubated for 24 h. Cells were harvested by washing with ice-cold PBS and then scraped in ice-cold lysis buffer (50 mM Tris, 150 mM NaCl, 1 mM EDTA, 1% Triton X-100, 0.1% SDS, 0.5% sodium deoxycholate, 5 mM NaF, 1 mM β-glycerolphosphate, 1 mM NaVO_3_ and 1/200 protease inhibitor cocktail (Sigma, P8340), pH 8.0). After sonication, cell debris was removed by centrifugation, and the supernatant was stored at −80°C. Protein concentrations were measured by the Bradford assay (Bio-Rad, Hercules, CA, USA) and the samples were used for Western blot analysis. All cell culture experiments were run in triplicates and all experiments were replicated.

### Western blotting

Western blot analysis was performed as previously described [24]. Briefly, frozen gastrocnemius muscle was homogenized in Mueller buffer and protein concentration determined by the Bradford method [27]. Crude muscle homogenate 40 μg was fractionated on 8% to 10% SDS-polyacrylamide gels. Gels were transferred to PVDF membranes overnight. Membranes were Ponceau stained to verify equal loading of each gel. Membranes were blocked overnight in 5% milk in Tris-buffered saline with 0.1% Tween-20 (TBS-T). Primary antibodies for CoxIV, Cytochrome C, Atg5, Beclin-1, LC3β, GAPDH and FoxO (Cell Signaling, Danvers, MA, USA), Mfn1, Mfn2 (Novus Biologicals, Littleton, CO, USA), Fis1 (Sigma), PGC-1α (Santa Cruz Biotechnology, Santa Cruz, CA, USA), p-FoxO (Millipore, Billerica, MA, USA) and 4-hydroxynonenal (alpha diagnostics) were diluted 1:1,000 to 1:500 in 5% milk in TBS-T followed by 1 h incubation with membranes at room temperature. Anti-rabbit or mouse IgG horseradish-peroxidase conjugated secondary antibodies (Cell Signaling, Danvers, MA, USA) were incubated with the membranes at 1:2,000 dilutions for 1 h in 5% milk in TBS-T. Enhanced chemiluminescence (ECL) (GE Healthcare Life Sciences, Piscataway, NJ, USA) was used to visualize the antibody-antigen interactions. Images were digitally scanned and blots were quantified by densitometry using scientific imaging software (Scion Image, Frederick, MD, USA).

### RNA isolation, cDNA synthesis, and real time PCR

RNA isolation, cDNA synthesis and real-time PCR were performed as previously described [28], using reagents from Applied Biosystems (Foster City, CA, USA). Fluorescence labeled probes for C2 proteasomal subunt, C7 proteasomal subunit, atrogin-1, Bax (FAM dye) and the ribosomal RNA 18 s (VIC dye) were purchased from Applied Biosystems and quantified with TaqMan Universal mastermix (Applied Biosystems, Foster City, CA, USA) PGC-1 (forward- 5′ AAGACGGATTGCCCTCATTT 3′, reverse 5′ AGTGCTAAGACCGCTGCATT 3′) and GAPDH primers were purchased from IDT (Coralville, Iowa, USA) and run using SYBR green PCR buffer. Data were analyzed by ABI software using the cycle threshold (C_T_), which is the cycle number at which the fluorescence emission is midway between detection and saturation of the reaction.

### Transmission electron microscopy

Samples of red quadriceps muscle were fixed in 2.5% glutaraldehyde and prepared as previously described [29]. Mitochondrial size was determined by tracing the outline of mitochondria at 15,000X magnification using Image J software (NIH, Bethesda, MA, USA).

### Statistical analysis

A one-way ANOVA was used to determine differences between *Apc*^*Min/+*^ mice separated by percentage body weight loss and all cell culture experiments. A two-way ANOVA was used to determine differences among variables in the IL-6 receptor antibody and exercise experiments. Post-hoc analyses were performed with Student Newman-Keuls methods. Significance was set at P <0.05.

## Results

**Figure 1:**
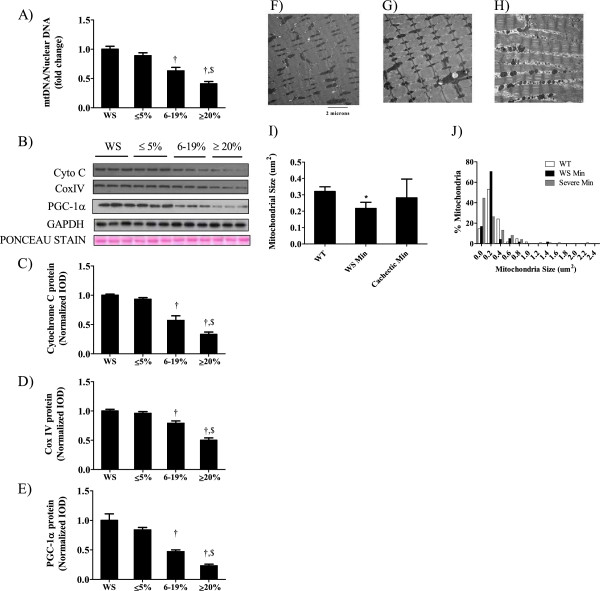
** Mitochondrial content, biogenesis and morphology are altered during the progression of cachexia.***Apc*^*Min/+*^ mice were grouped by percentage of body weight loss to study muscle oxidative capacity during the progression of cachexia. A) Mitochondrial content as determined by the mitochondrial:nuclear DNA ratio. B) Representative Western blot of cytochrome C, CoxIV and PGC-1α protein expression throughout the progression of cachexia. C) Cytochrome C, D) CoxIV and E) PGC-1α protein expression normalized to weight stable mice. Representative EM images of intramuscular mitochondria in F) wild-type, G) *Apc*^*Min/+*^ mice with mild cachexia and H) *Apc*^*Min/+*^ mice with severe cachexia. I) Mitochondrial size and J) mitochondrial size distribution. Values are means ± SE. Significance was set at P <0.05. † Signifies different from weight stable groups. $ Signifies difference from mice with 6 to 19% body weight loss. WS, weight stable.

Mitochondrial loss during severe cachexia is associated with a reduction in biogenesis and alterations in fission/fusion dynamics. *Apc*^*Min/+*^ mice were sacrificed between 14 and 20 weeks of age and then categorized as having no weight loss (weight stable), ≤5% body weight loss (initial), 6 to 19% weight loss (intermediate) and >20% loss (severe). While muscle mitochondria content was not different between weight stable mice and those exhibiting initial body weight loss, there was a 45% reduction (P = 0.03, Figure 1A) during intermediate body weight loss and a further reduction (63%; P = 0.005) with severe weight loss. Mitochondrial protein expression mirrored mitochondria content, with cytochrome C and Cox IV protein expression being reduced by 43% (P = 0.002; Figure 1C) and 21% (P = 0.002; Figure 1D) with intermediate weight loss and having expression of both proteins further reduced with severe weight loss. PGC-1α, a marker of mitochondrial biogenesis was reduced 53% (P = 0.003; Figure 1E) during intermediate stage cachexia and reduced further with the progression to severe body with loss (P = 0.002). The changes in mitochondrial protein expression and protein expression related to fission/fusion are associated with altered mitochondrial morphology in skeletal muscle. Electron microscopy images of skeletal muscle from wild-type (Figure 1F), weight stable *Apc*^*Min/+*^ mice (Figure 1G) and severely cachectic *Apc*^*Min/+*^ mice (Figure 1H). Mitochondrial size was reduced in weight stable *Apc*^*Min/+*^ mice compared to wild-type mice (Figure 1I). Mitochondrial size in cachectic *Apc*^*Min/+*^ mice was highly variable; however, when plotted as percentage mitochondrial size distribution there was a shift towards smaller mitochondria (Figure 1J) in cachectic *Apc*^*Min/+*^ mice when compared to weight stable *Apc*^*Min/+*^ mice and wild-type mice.

**Figure 2:**
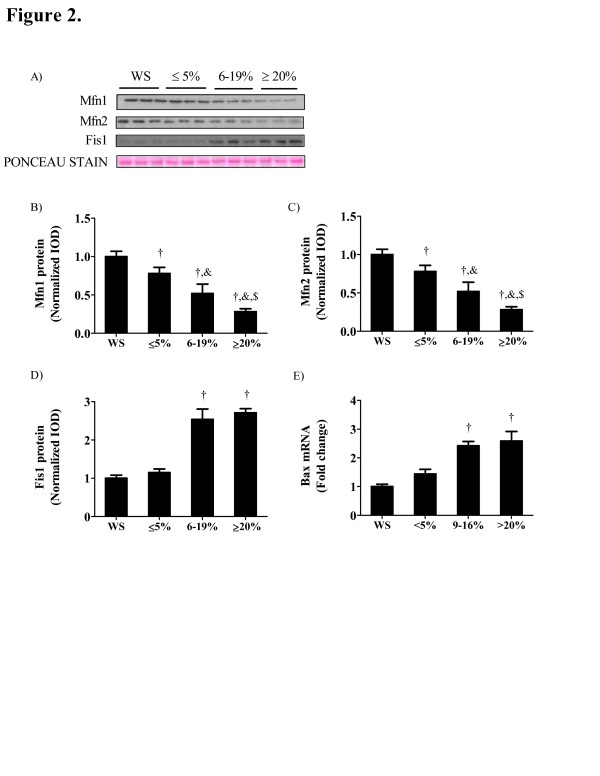
** Mitochondrial dynamics are altered during the progression of cachexia.** A) Representative Western blot of Mfn1, Mfn2 and FIS1 protein expression during the progression of cachexia. B) Mfn1, C) Mfn2 and D) FIS1 protein expression normalized to weight stable mice. E) Bax mRNA expression normalized to weight stable mice. Values are means ± SE. Significance was set at P <0.05. † Signifies the difference from WS groups. & Signifies the difference from mice with ≤5% body weight loss. $ Signifies the difference from mice with 6 to 19% body weight loss. WS, weight stable.

Mitochondrial fission/fusion proteins are differentially expressed during the progression of cachexia. Contrasting with muscle mitochondria content, the expression of mitofusin 1 (Mfn1) and Mfn2 proteins were reduced 22 and 31% (P = 0.04; Figure 2B, C) with the initiation of weight loss. With the progression of weight loss, muscle MFN1 and MFN2 expression was further reduced. There was no change in mitochondrial fission protein (FIS1) expression between weight stable mice and those having initial body weight loss, but FIS1 expression was strongly induced 2.5-fold (P = 0.002; Figure 2D) with the progression of body weight loss. Pro-apoptotic Bax mRNA expression was increased in *Apc*^*Min/+*^ mice with intermediate and severe body weight loss when compared to weight stable *Apc*^*Min/+*^ mice while no differences were detected in *Apc*^*Min/+*^ mice showing initial body weight loss (Figure 2E).

**Figure 3:**
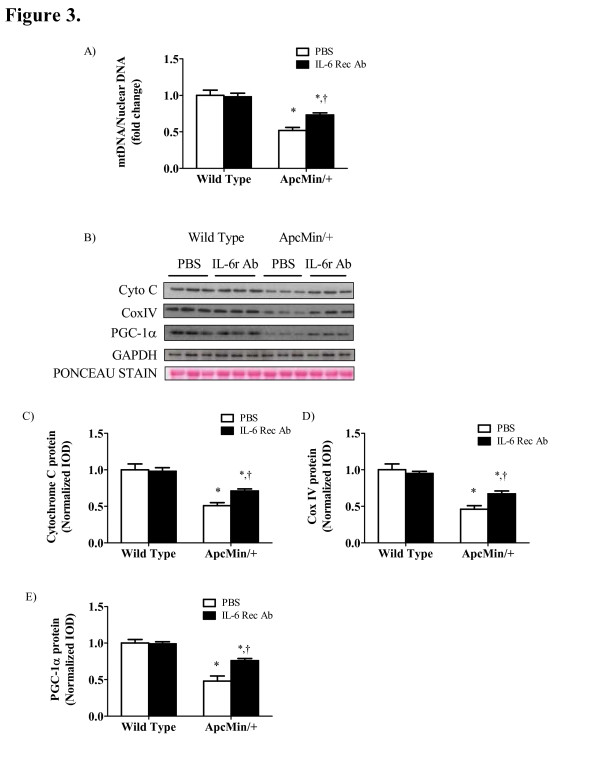
** IL-6 inhibition attenuates the loss in mitochondrial content and biogenesis in the*Apc***^***Min/+***^**mouse.** Wild-type and *Apc*^*Min/+*^ mice were treated with an IL-6 receptor antibody for two weeks to inhibit IL-6 signaling. A) Mitochondrial content as determined by the mitochondrial:nuclear DNA ratio. B) Representative Western blot of Cytochrome C, Cox IV and PGC-1α protein in wild-type and *Apc*^*Min/+*^ mice treated with an IL-6 receptor antibody or PBS control. C) Cytochrome C, D) CoxIV and E) PGC-1α protein expression normalized to wild-type PBS control mice. Values are means ± SE. Significance was set at P <0.05. *Signifies the difference within treatment. † Signifies the difference within genotype.

IL-6 inhibition attenuated mitochondrial loss in *Apc*^*Min/+*^ mice that have initiated body weight loss. We have previously reported inhibition of IL-6 signaling can attenuate the progression of cachexia and subsequent loss of muscle mass [1]. Here we show that the preservation of muscle mass is associated with the maintenance of mitochondrial biogenesis and dynamics. Control *Apc*^*Min/+*^ mice treated with PBS had a 59% reduction in mitochondrial content (P = 0.01; Figure 3A) and a reduction in cytochrome C and Cox IV and protein expression (P = 0.003; Figure 3C, D) when compared to wild-type controls, respectively. Inhibition of systemic IL-6 signaling by an IL-6 receptor antibody for two weeks attenuated the loss of mitochondrial content and repressed expression of mitochondrial proteins (Figure 3A, C, D). However, mitochondrial content and protein expression remained reduced compared to wild-type controls. Furthermore, IL-6 receptor antibody treatment attenuated the reduction in PGC-1α protein expression (P = 0.002; Figure 3E).

**Figure 4:**
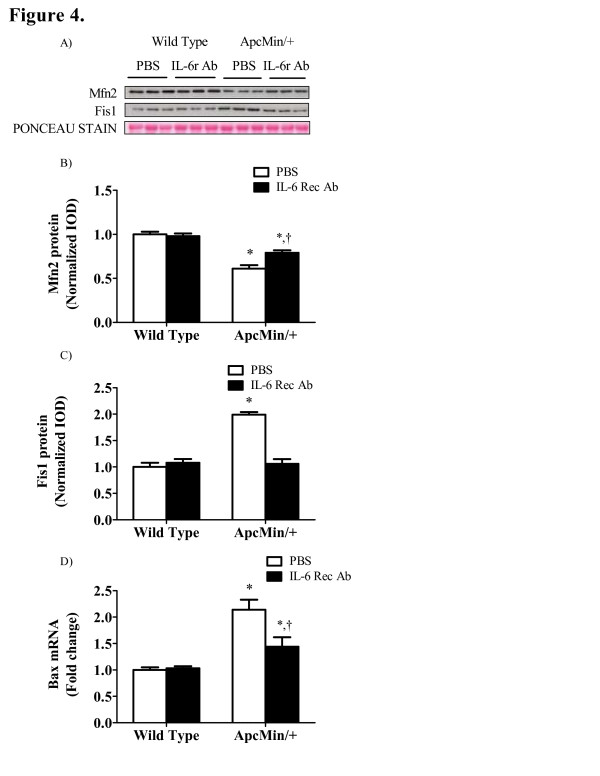
** IL-6 inhibition restores mitochondrial dynamics and reduces apoptosis in the*Apc***^***Min/+***^**mouse.** A) Representative Western blot of Mfn2 and FIS1 protein expression in wild-type and *Apc*^*Min/+*^ mice treated with an IL-6 receptor antibody or PBS control. B) Mfn2 and C) FIS1 protein expression normalized to PBS treated wild-type mice. D) Bax mRNA expression normalized to PBS treated wild-type mice. Values are means ± SE. Significance was set at P <0.05. * Signifies the difference within treatment. † Signifies the difference within genotype.

IL-6 inhibition attenuates the loss of mitochondrial fusion and prevents the expression of fission protein expression. Mfn2 protein expression was reduced 39% (P<0.001; Figure 4B) in PBS treated *Apc*^*Min/+*^ mice when compared to wild-type controls. IL-6 receptor antibody treatment increased Mfn2 expression in *Apc*^*Min/+*^ mice (Figure 4B), but not to wild-type levels. FIS1 protein expression was induced 2-fold (P <0.001; Figure 4C) in PBS treated *Apc*^*Min/+*^ mice and this induction was prevented by IL-6r antibody administration. The IL-6r receptor antibody did not alter muscle Mfn2 or FIS1 expression in wild-type mice. Bax mRNA expression was increased two-fold in PBS treated *Apc*^*Min/+*^ mice (Figure 4D) which was reduced 33% (P = 0.02) with IL-6 receptor antibody treatment.

**Figure 5:**
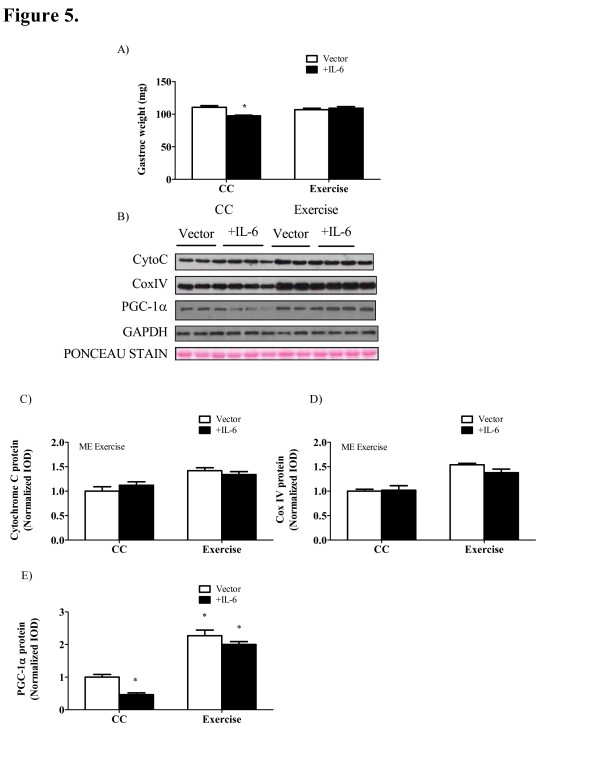
** IL-6 over-expression induced muscle wasting and reduced PGC-1α which are rescued with exercise training.***Apc*^*Min/+*^ mice underwent 12 weeks of moderate exercise training or served as sedentary cage controls and over-expressed circulating IL-6 or received a control vector. A) Gastrocnemius muscle mass. B) Representative Western blot of cytochrome C, CoxIV and PGC-1α protein in the gastrocnemius of *Apc*^*Min/+*^ mice. C) Cytochrome C, D) CoxIV and E) PGC-1α protein expression normalized to sedentary mice treated with the control vector. F) Upper - representative Western blot of 4-hydroxynonenal (4HNE)-modified proteins; lower - 4HNE-modified protein expression normalized to sedentary control mice. Values are means ± SE. Significance was set at P <0.05. CC, cage control; ME, main effect;. Wt, wild-type. * Signifies the difference from cage control vector mice.

IL-6-induced muscle wasting and associated alterations in mitochondrial dynamics are rescued with exercise training. Two weeks of IL-6 over-expression reduced gastrocnemius muscle mass by 12%, which was prevented when mice were exercise training during IL-6 over-expression (Figure 5A). Systemic IL-6 over-expression was not sufficient to alter mitochondrial protein expression in the gastrocnemius of *Apc*^*Min/+*^ mice initiating cachexia (Figure 5C, D). However, IL-6 over-expression decreased PGC-1α protein expression 56% (Figure 5E) in *Apc*^*Min/+*^ mice. In contrast, IL-6 over-expression did not decrease PGC-1α protein expression in exercise trained *Apc*^*Min/+*^ mice. Lastly, IL-6 over-expression or exercise training did not affect muscle oxidative damage as represented by quantification of 4-hydroxynonenal (4HNE)-modified proteins (Figure 5F).

**Figure 6:**
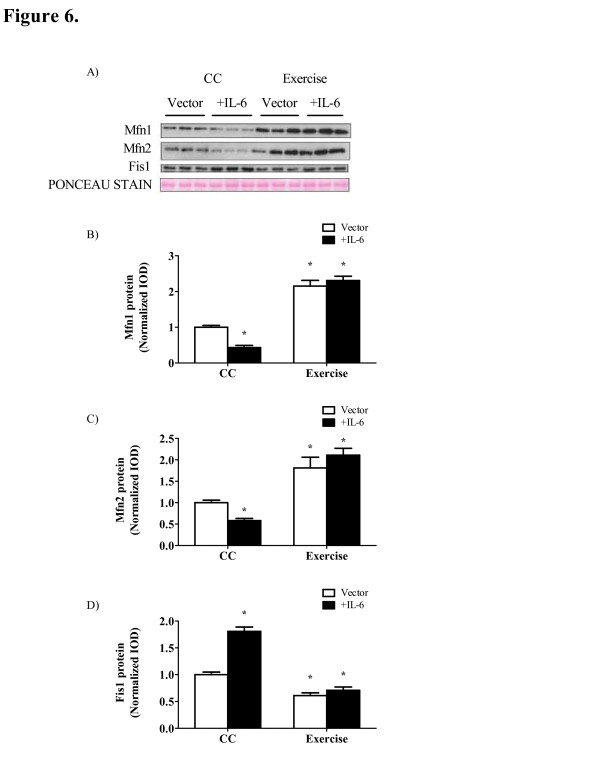
** Exercise training reduces IL-6 induced alterations in mitochondria dynamics, apoptosis and FoxO phosphorylation.** A) Representative Western blot of Mfn1, Mfn2 and FIS1 protein in the gastrocnemius of *Apc*^*Min/+*^ mice. B) Mfn1, C) Mfn2 and D) FIS1 protein expression normalized to sedentary mice treated with the control vector. E) upper - Representative Western blot of total and phosphorylated FoxO proteins; lower - ratio of phosphorylated to total FoxO protein expression normalized to cage control mice. F) Bax mRNA expression. Data are normalized to cage control mice. Values are means ± SE. Significance was set at P <0.05. CC, cage control; ME, main effect; Wt, wild-type. *Signifies the difference from cage control vector mice.

Exercise training improves IL-6 induced alterations in mitochondrial dynamic and apoptosis. Exercise is a potent method to increase oxidative capacity in skeletal muscle [7], and we have recently shown exercise can counteract muscle loss during IL-6-induced cachexia [22]. IL-6 over-expression decreased mitochondrial fusion proteins Mfn1 and Mfn2 57% and 42%, respectively (Figure 6B, C). Exercise was able to increase fusion protein expression by roughly two-fold despite IL-6 over-expression. Mitochondrial fission protein FIS1 was increased 81% with IL-6 over-expression which was prevented by exercise (Figure 6D). Phosphorylation of FoxO, a potent regulator of muscle proteolysis was decreased 44% (P = 0.003; Figure 6E) indicating increased transcriptional activation with IL-6 over-expression. Exercise training prevented the reduction in FoxO phosphorylation independent of circulation IL-6 levels. Bax mRNA expression was increased roughly two-fold (Figure 6F) with IL-6 over-expression which was also prevented by exercise training.

**Figure 7:**
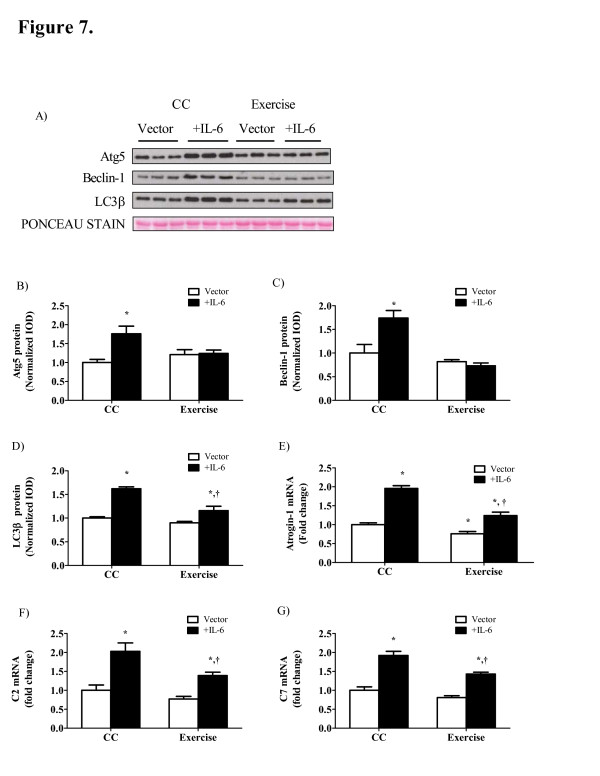
** Autophagy and ubiquitin dependent proteolysis are induced with IL-6 over-expression and attenuated with exercise training.** A) Representative Western blot of Atg5, Beclin1 and LC3β protein in the gastrocnemius of *Apc*^*Min/+*^ mice. B) Atg5, C) Beclin1 and D) LC3β protein expression normalized to sedentary cage control mice. E) Atrogin1 mRNA. F) C2 and G) C7 proteasomal subunit mRNA. Data are normalized to cage control groups for wild-type and *Apc*^*Min/+*^ mice. Values are means ± SE. Significance was set at P <0.05. *Signifies the difference from cage control vector mice. † Signifies the difference within IL-6 treatment.

IL-6 over-expression in *Apc*^*Min/+*^ mice increased muscle proteolysis through both ubiquitin dependent and autophagy related pathways. Autophagy related protein expression was increased with IL-6 over-expression as Atg5, Beclin-1 and LC3β were increased by 76%, 74% and 62% (P <0.05; Figure 7B, C, D) respectively. Exercise training prevented the induction of Atg5 and Beclin-1 and attenuated the induction of LC3β by 28% (P = 0.008). IL-6 over-expression induced gene expression related to the ubiquitin proteasomal pathway as well. The muscle specific E3 ligase, Atrogin-1 mRNA expression was induced by two-fold (Figure 7E) while mRNA expression of proteasomal subunits C2 (Figure 7F) and C7 (Figure 7G) were increased roughly two-fold. Exercise training attenuated gene expression related to ubiquitin dependent proteolysis.

**Figure 8:**
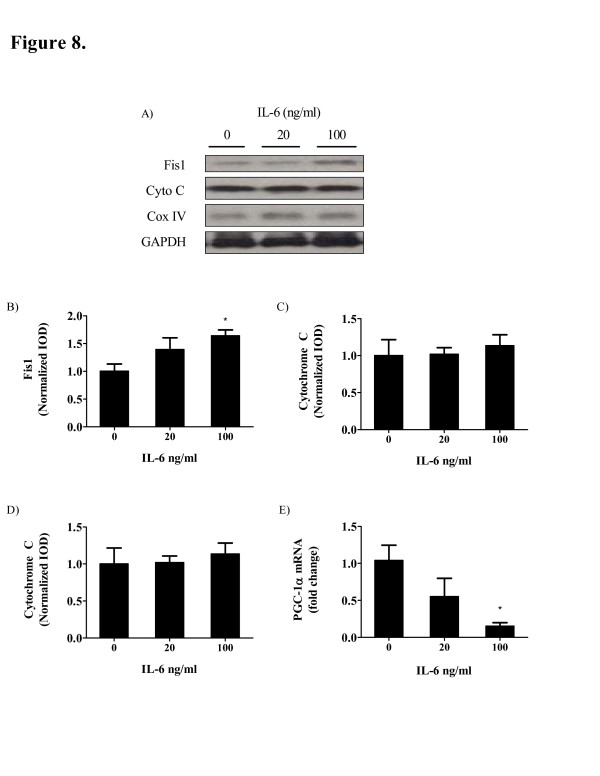
** IL-6 treatment on C2C12 myotubes induces FIS1 expression and a reduction in PGC-1α mRNA.** A) Representative Western blot of FIS1, Cytochrome C and CoxIV protein in C2C12 myotubes treated with 0, 20 and 100 ng/ml of IL-6. B) FIS1, C) Cytochrome C and D) CoxIV C protein expression and PGC-1α mRNA E) expression normalized to C2C12 myoblasts treated with vehicle control (0 ng/ml IL-6). Values are means ± SE. Significance was set at P <0.05. *Signifies the difference from 0 ng/ml control.

IL-6 treatment to C2C12 myotubes induces Fis1 and oxidative damage independent of changes in PGC-1α and mitochondrial proteins. C2C12 myotubes were treated with 0 ng/ml IL-6 (Control), 20 ng/ml (low) and 100 ng/ml (high). A total of 100 ng/ml of IL-6 induced Fis1 protein expression 64% (P = 0.02; Figure 8B) when compared to control while no change in Fis1 expression was detected in the low dose of IL-6. The low dose of IL-6 trended (P = 0.07) to decrease PGC-1α mRNA expression while the high IL-6 dose decreased PGC-1α mRNA expression by roughly 80% (P <0.001; Figure 8E) without any changes in mitochondrial proteins cytochrome C (Figure 8C) and Cox IV (Figure 8D). 4HNE modified proteins were increased 43% (P = 0.04; Figure 8F) with the high dose of IL-6 when compared to control. The low dose of IL-6 did not affect 4HNE modified proteins in C2C12 myotubes.

## Discussion

Skeletal muscle mitochondria have emerged as a critical regulator of muscle protein turnover [5]. Both mitochondrial loss and altered fission/fusion regulation are associated with muscle wasting [30-32]. Related to these processes are induction of reactive oxygen species, apoptosis and activation of the ubiquitin and autophagy dependent proteolysis [5]. We have previously shown a reduction in muscle oxidative capacity and altered mitochondrial dynamics in both oxidative and glycolytic muscle in severely cachectic *Apc*^*Min/+*^ mice [9]. In addition, we have recently reported the induction of both ubiquitin and autophagy related muscle protein degradation during the progression of cachexia [1]. Here we report the novel finding that the expression of proteins regulating mitochondrial biogenesis and mitochondrial dynamics are disrupted early in the development of cachexia and precede a reduction in mitochondria content. Further, alterations in the expression of these proteins can be suppressed by the administration of an IL-6r antibody after the initiation of cachexia. We also report that exercise training can improve the expression of proteins regulating mitochondrial biogenesis and dynamics, which is associated with the attenuation of muscle protein degradation even when systemic IL-6 levels are comparable to what is typically observed during severe cachexia. Lastly, we show IL-6 treatment to C2C12 myotubes induced FIS1 expression and oxidative damage without changes in oxidative protein expression.

While we previously reported a reduction in mitochondrial content and protein expression in severely cachectic *Apc*^*Min/+*^ mice [9], that study was not able to examine changes during the progression of the disease. Our current study expanded on these prior findings by stratifying *Apc*^*Min/+*^ mice into groups of incremental weight loss. We report that the loss of muscle mitochondria is not necessary for the significant amount of muscle mass loss (approximately 20%) that occurs at the onset of cachexia. However, there was an incremental loss of skeletal muscle mitochondria with further progression of cachexia, which coincides with the induction of apoptosis in the muscle [10], and the induction of proteins regulating autophagy [1]. We report the novel finding that muscle mitochondrial morphology is altered during the initiation and progression of cancer cachexia. Late stage cachexia in *Apc*^*Min/+*^ mice is also associated with a surge in circulating IL-6 and a reduction in volitional physical activity [12]. While our current study reports that two weeks of elevated circulating IL-6 was not sufficient to reduce muscle mitochondrial content, the IL-6r antibody treatment after the initiation of cachexia was able to significantly attenuate the loss of mitochondria. Skeletal muscle mitochondrial content retains plasticity related to the amount of contractile activity being performed by the muscle [33]. Here we also show that exercise training prior to and during over-expression of IL-6 in the *Apc*^*Min/+*^ mouse could not only prevent the suppression of mitochondrial biogenesis, but increase oxidative protein expression above control values regardless of cachectic stimuli. Further work is needed to understand the association between sedentary behavior and chronically high IL-6 levels, which are characteristics of late stage cachexia, on the processes regulating mitochondria loss during the progression of cachexia.

The suppression of mitochondria biogenesis during the initiation of cachexia could be a critical early event that leads to mitochondrial dysfunction and loss in later stages of the disease. Interestingly, the reduction in mTOR signaling, and specifically the mTORC1 complex, in cachectic muscle may impact mitochondrial content through the repressed transcription of genes involved in oxidative metabolism. The mTORC1 complex can act with PGC-1α to activate transcription of oxidative genes [34], and muscle mitochondria content is severely reduced in mice with a muscle specific RAPTOR knockout, which disrupts the formation of the mTORC1 complex [35]. We have reported the phosphorylation of RAPTOR, which decreases mTORC1 activation, is increased during the progression of cachexia in the *Apc*^*Min/+*^ mouse [1]. PGC-1α has a well-documented role in the regulation of skeletal muscle oxidative capacity [36,37] and, recently, it has been shown to be involved with numerous cellular processes including protein degradation [38], mTOR activity [34], apoptosis [39] and regulation of ROS [40]. A reduction in PGC-1α expression has been previously shown in several wasting diseases [41-45], including cachexia [9]. The reduction in muscle PGC-1α expression coincided with circulating IL-6 levels, being repressed by IL-6 over-expression before a reduction in oxidative capacity, and being further reduced with the progression of cachexia. Additionally, the administration of the IL-6r antibody attenuated the loss in PGC-1α expression and exercise served to induce PGC-1α expression in muscle that demonstrated an attenuated catabolic response related to muscle wasting. Furthermore, we report IL-6 can have a direct effect on mitochondria biogenesis as C2C12 myotubes treated with IL-6 resulted in a reduction in PGC-1α mRNA expression. Therefore, induction of mitochondrial biogenesis during the initiation of cachexia may be an excellent target for therapeutic intervention during the initiation of cachexia.

The coordination of mitochondrial fission and fusion, referred to as mitochondrial dynamics, have been recently identified as critical components of mitochondrial function, morphology and distribution [6,46]. Fusion proteins Mfn1 and Mfn2 can promote mitochondrial elongation and activity, and regulate mitochondrial membrane potential and glucose oxidation in cultured cells [47]. We report that the expression of both Mfn1 and Mfn2 proteins are repressed during pre-cachexia, and this is one of the earliest alterations in protein expression related to oxidative metabolism we have found in skeletal muscle. There is a further reduction in Mfn1 and Mfn2 protein expression as cachexia progresses. Mitochondrial fusion protein expression appears to be IL-6 sensitive. It has been previously shown that IL-6 treatment to muscle cells reduced mitochondrial fusion protein Mfn2 gene expression [17]. In the current report, IL-6 over- expression repressed expression and IL-6r antibody administration increased expression of Mfn2. Interestingly, exercise was able to increase both Mfn1 and Mfn2 expression regardless of IL-6 levels. PGC-1α and PGC-1β can regulate Mfn2 gene expression in conjunction with the estrogen-related receptor-α [3,48]. A reduction in Mfn2 has been observed in muscle from type 2 diabetic [17,49] and obese patients [17]. However, patients with chronic heart failure and weight loss did not show a change in muscle Mfn2 indicating the role of Mfn2 in muscle wasting may be different depending on disease type and severity [50].

Related to the regulation of mitochondrial fission, our data show an increase in muscle FIS1 protein expression in the later stages of cachexia, which could also be induced by systemic over-expression of IL-6. Furthermore, we are the first to show that FIS1 gene expression in C2C12 myotubes is increased when treated with IL-6. The role of FIS1 during wasting diseases is currently unknown, but FIS1 over-expression is pro- apoptotic in skeletal muscle [51-53] and is associated with the production of reactive oxygen species [5] and activation of muscle protein degradation [32]. Muscle apoptosis is commonly observed during cancer cachexia [10,54,55]. We have previously shown the induction of apoptosis in muscle from severely cachectic *Apc*^*Min/+*^ mice while no evidence of apoptosis was observed in moderately cachectic *Apc*^*Min/+*^ mice [10]. Here we show FIS1 was only increased during later stages of cachexia, which coincided with the induction of pro-apoptotic Bax mRNA expression, which further suggests an association between mitochondrial fission and apoptosis. Furthermore, exercise training was able to reduce fission protein and Bax mRNA expression. To determine whether the exercise-induced suppression of fission and apoptosis coincided with inhibition in muscle degradation, we measured markers of autophagy and ubiquitin dependent proteolysis. We report exercise was able to attenuate activation of FoxO and pathways related to both autophagy and the ubiquitin proteasomal system. This effect was similar to what was observed by Romanello et al. when they showed inhibition of mitochondrial fission prevented muscle wasting induced by starvation or FoxO over-expression [32]. Together, these findings suggest improvements in mitochondrial dynamics could be a target for exercise-induced protection from muscle protein degradation and eventual muscle wasting.

The production of reactive oxygen species is associated with mitochondrial remodeling and activation of proteolytic pathways during muscle wasting [5]. The role of reactive oxygen species during muscle wasting in *Apc*^*Min/+*^ mice is still unclear. We previously reported no changes in muscle oxidative damage during cachexia in the *Apc*^*Min/+*^ mouse [9] and currently show no changes in oxidative damage with systemic IL-6 over-expression. However, IL-6 treatment on C2C12 myotubes increased indices of reactive oxygen species. Further investigation is needed to determine the role of ROS production in regulating muscle wasting during cachexia.

## Conclusions

In summary, we show the reduction in mitochondrial content during the progression of cachexia in the *Apc*^*Min/+*^ mice occurs during later stages of body weight loss. The loss of mitochondria is preceded by the reduction in PGC-1α and mitochondrial fusion proteins Mfn1 and 2 during the initial stages of cachexia, while the induction of fission protein FIS1 occurs with the progression of cachexia. In this study, we used an IL-6 receptor antibody to inhibit IL-6 signaling after the initiation of cachexia, used systemic IL-6 over-expression to initiate cachexia, and also examined the effect of exercise to improve muscle mitochondrial function during IL-6 induced cachexia. Lastly, C2C12 myoblasts were treated with IL-6 to determine direct effects of IL-6 on mitochondrial remodeling/function. We have shown IL-6 inhibition and exercise training can prevent the progression of cachexia in *Apc*^*Min/+*^ mice. Here, we show both therapies can prevent the loss of mitochondrial content by preserving PGC-1α and fusion protein expression, while preventing the increase in fission protein expression. These changes were associated with a reduction in muscle wasting and pathways related to muscle protein degradation. Our in vitro data extended our finding by showing IL-6 can directly increase FIS1 expression in muscle cells. Further research needs to examine if therapies to maintain muscle oxidative capacity are most effective when administered before significant body weight loss develops. The findings of this study enhance our understanding of the interactions between muscle oxidative capacity and the regulation of muscle mass during cachexia, thus, providing further rationale to explore the interconnection between oxidative capacity and its role in regulating muscle mass during wasting diseases.

## Abbreviations

FIS1, Mitochondrial fission protein1; MFN1, Mitofusin1; MFN2, Mitofusin2; PGC-1α, Peroxisome proliferator-activated receptor-gamma co-activator 1 alpha.

## Competing interest

The authors declare that they have no competing interests.

## Authors’ contributions

JPW, JWB, MJP, MCK, LEM, SS and JAC conceived and designed the experiments. JPW, MJP, SG, SS and JAC performed the experiments. JPW, JWB, MJP, SG, MCK, LEM, SS and JAC analyzed the data. JPW, JWB, MCK, LEM and JAC contributed reagents, materials and analysis tools. JPW and JAC wrote the paper. LTD, JPW and JAC obtained use of the IL-6 receptor antibody from Chugai Pharmaceutical Company. All authors read and approved the final manuscript.
